# Improving District Hospital Surgical Capacity in Resource Limited Settings: Challenges and Lessons From South Africa: Comment on "Improving Access to Surgery through Surgical Team Mentoring – Policy Lessons From Group Model Building with Local Stakeholders in Malawi"

**DOI:** 10.34172/ijhpm.2022.6937

**Published:** 2022-02-14

**Authors:** Lynn Bust, Kathryn Chu

**Affiliations:** ^1^Centre for Global Surgery, Department of Global Health, Stellenbosch University, Cape Town, South Africa.; ^2^Department of Surgery, University of Botswana, Gaborone, Botswana.

**Keywords:** District Hospital, Mentoring, Low-Resource Setting, South Africa, Surgery

## Abstract

Strengthening surgical capacity of district hospitals (DHs) in low- and middle-income countries (LMICs) has been recognised globally as key to improving equitable access to surgical care. This commentary considers the benefits and challenges of surgical mentoring in South Africa and applies the lessons learned to other low-resource settings. Surgical team mentoring programmes require consideration of all stakeholders involved, with strong relationships between mentors and mentees, and the possible establishment of roaming district surgical teams. Other components of a surgical ecosystem must also be strengthened including defining a DH surgical package of care, ensuring strong referral systems through a hub and spoke model, and routine monitoring and evaluation. These recommendations have the potential to strengthen surgical capacity in DHs in low-resource settings which is critical to achieving health for all.

## Introduction

 The article by Broekhuizen et al on improving access to surgery through surgical team mentoring in Malawi contributes to an increasingly important discussion about strengthening the surgical capacity of district hospitals (DHs) in low- and middle-income countries (LMICs).^1^ Task sharing, or the delegation of some tasks to cadres with less training, was a key premise of the surgical mentorship programme in Malawi. In task sharing, fully trained surgeons can teach, mentor, and supervise other cadres such as mid-level providers, doctors, and nurse practitioners with surgical skills. Implementation of task sharing should occur as a crucial part of scaling up of DH surgical capacity. Task sharing needs to be operationalised in LMICs, particularly in the countries in which there are a severe shortage of surgeons.

 The study by Broekhuizen et al provides important lessons for the sustainment and national roll-out of surgical team mentoring programmes.^1^ The aim of the programme was to empower DHs to undertake a larger range and number of surgical procedures thus improving access to surgical care. The study utilised group model building workshops to formulate causal loop diagrams which highlighted the complexity of surgical team mentoring. It found that routine monitoring and communication between mentees at DHs and surgical specialists is required. An output- or performance-based financing scheme for DHs was recommended to incentivise the scale up of surgery.

 Globally, there has been a growing focus on increasing access to surgical care, particularly through strengthening DH surgical capacity.^2^ In 2015, the World Health Assembly passed a declaration stating that DHs should be the backbone of essential and emergency surgical care (EESC), which was recognised as a key component of universal health coverage.^3^ To achieve equitable access to EESC, strengthening surgical health systems at the district level should be prioritised.^2^ Healthcare provided closer to the community has been shown to have improved outcomes, reduce patient out-of-pocket expenditure, and improve health-seeking behaviour.^4^ A higher level of EESC provided by DHs would not only improve equitable access for patients, but also decrease the burden on regional/tertiary hospitals.^5^ This is particularly relevant in regions with higher levels of inequity such as LMICs, and rural areas.

 South Africa is an LMIC with a highly inequitable health system. Historically, delivery of DH surgical care in this country has been limited, but more recent recognition of the importance of decentralisation of services to improve access to timely and quality surgical care has taken place.^6^ Challenges to increasing the capacity of rural surgical care in South Africa include lack of training, communication, and support from regional hospitals; skills-resource mismatch; and an ill-defined DH role in surgical provision.^2^ We consider some of the challenges identified in the South African context which could be applied when utilising strategies such as surgical team mentoring to strengthen DH surgical capacity in other LMICs.

## Stakeholder Considerations

 The implementation of a successful surgical team mentoring programme requires buy-in from all stakeholders involved including mentors, mentees, and management. Broekhuizen et al showed that surgical team mentoring had to be found acceptable by those involved, and motivation of both mentors and mentees was identified as a key component of the programme’s success.^1^ Baseline level of surgical knowledge of each cadre of healthcare provider should be assessed when designing mentorship programmes and establishing buy-in from stakeholders in various countries.

 Surgeons must believe in the concept of surgical mentoring, especially that the training and mentoring of other surgical cadres will lead to improved surgical access at rural DHs. However, there are several reasons why surgeons might be reluctant to become surgical mentors. Surgeons who have not bought in fully to the concept of task sharing might be reluctant and consider surgical care delivered by non-specialists to be inferior.^2^ In many LMICs, mid-level providers are critical in surgical care delivery. In Malawi, clinical officers are being taught to provide DH surgical care, and Mozambique has successfully institutionalized surgical assistants or ‘técnico de cirurgia.’^1,7^ There is a lack of evidence in LMIC settings of the knowledge, attitude and perceptions of task sharing amongst surgical providers which should be further investigated.

 Most LMICs have a shortage of public sector surgeons, most of whom work in urban regional/tertiary hospitals. Outreach to DHs directly competes with higher-level hospital service delivery especially if surgeons are away for more than one day a week. Broekhuizen et al suggested financial incentives for mentors as well as DH staff.^1^ This could be complicated to achieve in LMICs such as South Africa where public healthcare staff are on fixed government salaries. Top up for those going on outreach programmes might leave those left behind to staff regional/tertiary surgical services feeling unfairly disadvantaged. Further systemic challenges to providing top-ups in LMIC settings such as a lack of government funding should also be addressed.

 Buy-in from mentees at DHs is critical. In some LMICs such as South Africa, DH surgery is an add-on responsibility of non-surgeon doctors (medical officers and family physicians) who have non-surgical primary clinical responsibilities such as working on another medical ward or in the emergency department. The motivation and commitment for these doctors to learn surgical skills and be part of a mentoring programme would need to be individually assessed. In South Africa, family physicians receive select surgical training during their specialisation. Some already perform DH surgery and others have expressed a willingness to upskill but fear criticism from surgeons if complications arise.^6^ A mentoring programme would be a mutually beneficial way to provide support and establish trust between themselves and surgeon mentors. Whilst building relationships with family physicians may be beneficial, DHs in South Africa are often staffed by community service or junior doctors who are re-assigned after one year. A high turnover of mentees would unlikely make any mentoring programme successful. This was also found in Malawi where mentors found it hard to establish relationships with changing local teams.^1^

 Finally, buy-in from management at the district, provincial, and national level would be required for successful DH surgical mentoring. Broekhuizen et al recommends a national senior coordinator in Malawi.^1^ However, in other LMICs, this might be better coordinated at the district or provincial level depending on the health governance structure.

 Establishing a successful mentoring model would require support, coordination, and funding at the provincial and national level, as well as buy-in from mentors and mentees. An alternative mentoring model to the outreach programme proposed by Broekhuizen et al that would not compete for regional/tertiary hospitals’ human resources would be the establishment of district surgical teams.^1^ These teams would not have competing home hospital duties but rather only the mandate to mentor surgical staff at various DHs and ensure quality clinical surgical services at the DH level.

 South Africa already has a similar model for primary healthcare. Since 2012, District Clinical Specialist Teams (DCSTs) have been established to improve maternal and child health in rural health districts, amongst other activities.^8^ Each DCST has a paediatrician, family physician, obstetrician and gynaecologist, and anaesthetist, as well as an advanced midwife, advanced paediatric nurse, and advanced primary healthcare nurse. A multi-disciplinary approach, such as in the DCSTs, is needed in surgical mentoring as well. A comprehensive surgical training requires upskilling DH staff in anaesthesia as well as operative skills. In a recent study from the Western Cape province in South Africa, the lack of anaesthesia skills at DHs was a major barrier to upscaling DH surgery.^9^ The potential add-on of a surgeon to the DCST, which already contains a trained anaesthetist, could be explored as a substitute to stand-alone district surgical teams. For this model to be successful, surgery would need to be recognised as a priority on the national health agenda with substantial buy-in from the Ministry of Health.

 Regardless of the model, buy-in from all stakeholders requires establishment of relationships and trust. In the case of surgical team mentoring, building trust between mentors and mentees takes time and the investment of all parties. How these teams function together would influence surgical care provision at DHs. Relationships, or the ‘software’ of the health system (norms, traditions, values, roles and procedures),^10^ should be taken into consideration when implementing interventions to improve DH surgical capacity.

## Strengthening the Entire Surgical Ecosystem

 Surgical mentoring is a key component to strengthening surgical capacity at DHs, however, other components are also necessary. These include defining a DH surgical package; creating hub and spoke networks to ensure strong referral systems supported by telemedicine; and routine monitoring of surgical outcomes. These factors will complement a surgical team mentoring programme to strengthen the entire surgical ecosystem.

###  Defined District Hospital Surgical Packages

 Clearly defined DH surgical packages would help guide surgical mentoring programmes as well as strengthen surgical capacity more broadly. The World Health Organization (WHO) has described a list of 28 surgical procedures that should be performed at DHs.^3^ It is recommended that countries should adapt or determine their own surgical baskets of care based on available human resources and clinical needs.^11^ The South African National Department of Health has over 100 surgical procedures in its DH package of care,^12^ but few DHs can provide all of these. It would be more prudent to start with a smaller basket of DH surgical procedures. We suggest less than 20 to allow targeted training, as well as the ability to ensure the necessary equipment and supplies, with the opportunity to repeat training or expand at a later stage. In terms of surgical mentoring, a defined surgical package of care would help to determine the end goal of each mentorship, and guide both mentors and mentees in their training.

###  Hub and Spoke Model

 Patient-centred care and health system strengthening are better achieved when hospitals from different levels are interconnected.^13^ One challenge for DHs has been lack of efficient referrals to higher level hospitals when needed. In addition, once in-person surgical mentoring has been completed, DH surgical providers need a way to be supported by higher level hospital surgeons especially once they start operating without supervision. To address both these needs, we propose a hub and spoke model where “hub” regional/tertiary hospitals support a network of “spoke” DHs. All surgical providers need to be able to ask for advice and refer patients that become too complicated either pre- or post-operatively. The hub and spoke model ensures ongoing support even when the outreach or district teams are not physically present at the DH. These hub and spoke networks should form part of well-established, national referral systems.

 Telemedicine platforms can be utilised to provide advice and expedite referrals across hub and spoke hospitals. Broekhuizen et al utilise a remote WhatsApp clinical support network in conjunction with the surgical team mentoring visits.^1^ Mobile health tools such as WhatsApp or Vula Mobile (a mobile health referral app used for referrals in South Africa) have been shown to improve communication and could be used as teaching platforms in LMIC settings.^14,15^

###  Monitoring and Evaluation

 Monitoring and evaluation can be particularly challenging in low-resource settings. For example, hospitals often lack funding for routine data collectors to record surgical volume and outcomes. The paucity of internet, computer systems, and electronic medical records also make routine monitoring and evaluation more time consuming and difficult. The quality of DH surgical care can be strengthened by routine monitoring and evaluation using prospective operative databases to track patient outcomes. Hub and spoke networks should hold joint morbidity and mortality conferences to review expected and unexpected complications. This will allow continued mentoring, feedback, and follow-up of transferred patients as well as build the relationship between surgical providers at all hospital levels.

## Conclusion

 Globally, the importance of strengthening surgical capacity in DHs has been recognised.^3^ Broekhuizen et al provide useful insights into the establishment of a surgical team mentoring programme as a way to improve access to surgical care.^1^ However, strengthening surgical capacity in DHs is a complex process which requires the consideration of all interconnected parts of a surgical ecosystem. Lessons for surgical mentoring can be learnt from the South African context, but there are unique challenges within each surgical ecosystem that should be considered. Improving DH surgical capacity should not be a one-size-fits-all endeavour and should be context-specific.

 Adapting an intervention to local context is important, and several recommendations for strengthening surgical capacity in resource-limited settings have been discussed (summarised in Table). These inter-related factors include gaining buy-in from all stakeholders for surgical team mentoring, implementing a hub and spoke model, defining DH surgical care packages, ensuring availability of necessary equipment and human resources, and monitoring and evaluation. Finding innovative ways to improve provision of surgical care in DHs is crucial to achieving health for all.

**Table:** Strengthening District Hospital Surgical Health Capacity in Low- and Middle-Income Countries

**Recommendation**	**Explanation**
1. Surgical mentoring	Train lower-level surgical cadres to perform surgical care at rural DHs. Consider travelling district training teams as alternative model to outreach. Build trust and relationships between mentors and mentees.
2. Equipment and human resources	Ensure the necessary equipment and supplies are available. Provide surgery and anaesthetic training to all levels of providers to create well-equipped teams to ensure safe surgery.
3. Surgical packages of care	Clearly define DH surgical packages of care. Adapt baskets of care based on available human resources and clinical needs. Helps to define training and resource needs.
4. Hub and spoke networks	Link “spoke” DHs with “hub” referral hospitals to improve communication and referrals. Supplement with telemedicine tools. Promote co-management of patient care.
5. Monitoring and evaluation	Routine monitoring and evaluation through prospective databases. Morbidity and mortality conferences to improve quality of care and ensure continued mentoring of lower surgical cadres.

Abbreviation: DHs, district hospitals.

## Ethical issues

 Not applicable.

## Competing interests

 Authors declare that they have no competing interests.

## Authors’ contributions

 LB wrote first draft of the manuscript. KC and LB reviewed and contributed to final draft.
